# Oxygenation-sensitive CMR for assessing vasodilator-induced changes of myocardial oxygenation

**DOI:** 10.1186/1532-429X-12-20

**Published:** 2010-03-31

**Authors:** Matthias Vöhringer, Jacqueline A Flewitt, Jordin D Green, Rohan Dharmakumar, Jiun Wang, John V Tyberg, Matthias G Friedrich

**Affiliations:** 1 Stephenson Cardiovascular MR Centre at the Libin Cardiovascular Institute of Alberta, Department of Cardiac Sciences, University of Calgary, Calgary, AB, Canada; 2 Robert-Bosch-Krankenhaus, Stuttgart, Germany; 3 Siemens Healthcare, Erlangen, Germany; 4 Department of Radiology, Northwestern University, Chicago, IL, USA; 5 Fu-Jen University Medical School, Hsinchuang, Taiwan; 6 Department of Cardiac Sciences and Physiology/Pharmacology, University of Calgary, Calgary, AB, Canada; 7 Stephenson Cardiovascular MR Centre at the Libin Cardiovascular Institute of Alberta, Department of Cardiac Sciences, University of Calgary, Calgary, AB, Canada

## Abstract

**Background:**

As myocardial oxygenation may serve as a marker for ischemia and microvascular dysfunction, it could be clinically useful to have a non-invasive measure of changes in myocardial oxygenation. However, the impact of induced blood flow changes on oxygenation is not well understood. We used oxygenation-sensitive CMR to assess the relations between myocardial oxygenation and coronary sinus blood oxygen saturation (SvO_2_) and coronary blood flow in a dog model in which hyperemia was induced by intracoronary administration of vasodilators.

**Results:**

During administration of acetylcholine and adenosine, CMR signal intensity correlated linearly with simultaneously measured SvO_2 _(*r*^2 ^= 0.74, *P *< 0.001). Both SvO_2 _and CMR signal intensity were exponentially related to coronary blood flow, with SvO2 approaching 87%.

**Conclusions:**

Myocardial oxygenation as assessed with oxygenation-sensitive CMR imaging is linearly related to SvO_2 _and is exponentially related to vasodilator-induced increases of blood flow. Oxygenation-sensitive CMR may be useful to assess ischemia and microvascular function in patients. Its clinical utility should be evaluated.

## Background

Myocardial oxygenation, reflecting the balance or imbalance between oxygen demand and supply, is an important diagnostic target in various clinical settings[1], but may be especially useful for assessing ischemia and microvascular function. Presently available diagnostic tools are invasive, use exogenous contrast agents and/or radiation, are only useful in particular coronary territories, or have a limited spatial resolution[2,3]. Moreover, they do not provide direct measures of ischemia.

T_2_*-sensitive, Blood-Oxygen-Level-Dependent Cardiovascular MR (BOLD-CMR) uses the paramagnetic properties of deoxygenated hemoglobin as an endogenous contrast mechanism and is thus oxygenation-dependent[4]. In oxygenation-sensitive CMR images, the signal intensity of any soft tissue is inversely correlated with its absolute content of deoxygenated hemoglobin and is therefore theoretically sensitive to changes in blood volume and oxygen supply-demand balance[5]. Such sequences have been used routinely for functional brain imaging[6] and similar approaches have been applied to the heart and peripheral perfusion beds [7-10]. Although these T_2_* measurement and T_2_*-mapping techniques have been shown to have high BOLD sensitivity, they had limited clinical use thus far because of long acquisition times and relatively low signal-to-noise ratios. Additionally, magnetic field inhomogeneities, blood flow and cardiac motion may all impair image quality.

Recently, BOLD-sensitive, steady-state free precession (SSFP) techniques with much more consistent image quality have been introduced [11-13] and applied in experimental models of coronary artery stenosis[14,15]. To date, however, SSFP BOLD-CMR has not been validated against simultaneous measurements of myocardial oxygenation changes. Moreover, BOLD-weighted SSFP imaging has not been compared against other approaches such as T_2_* mapping.

We hypothesized that SSFP BOLD-CMR can accurately and consistently detect changes of myocardial oxygenation *in vivo*.

## Methods

We used a canine model with selective intracoronary vasodilator infusion. In order to cover the full range of physiological flow changes we applied graded infusions of the endothelium-dependent vasodilator, acetylcholine, as well as the endothelium-independent vasodilator, adenosine.

### Animal Preparation

Seven mongrel dogs (weight 15 to 25 kg) were studied; all experiments were conducted in accordance with the most recent policies and "Guide to the Care and Use of Experimental Animals" by the Canadian Council on Animal Care. The local animal care and use board approved the study protocol and sample size.

Under general anesthesia, a midline sternotomy was performed and a 2-mm MR-compatible flow probe (Transonic Systems Inc., Ithaca, NY) placed around the proximal left circumflex (LCX) coronary artery. Under fluoroscopic control, a 2.7-F infusion catheter (Tracker^®^-18 Hi-Flow, Boston Scientific Ltd., Cork, Ireland) was introduced into the LCX through a diagnostic coronary catheter (JL 2.5, Torcon NB ^® ^Advantage Catheter, Cook ^®^, Denmark). The tip of the infusion catheter was placed a few millimeters proximal to the flow probe while ensuring that there were no visible side branches located between the infusion catheter and flow probe. In addition, a 4-F balloon catheter (Berman Angiographic Balloon Catheter, Arrow, Reading, PA, USA) was introduced into the coronary sinus (CS) for blood sampling. Blood gases were analyzed using a portable analyzer (STAT PROFILE^® ^Critical Care Xpress, Nova Biomedical, Waltham, MA, USA). All procedures including the CMR scan were performed in adjacent rooms with the dogs being placed on an MR-compatible cradle that allowed for a quick and easy transport to and from the MR system.

### CMR Protocol

All CMR scans were performed in a clinical 1.5-T MRI system (MAGNETOM Avanto^®^, Siemens Healthcare, Erlangen, Germany) with a 6-element phased-array coil resting on the chest and another below the spine. After acquiring localizer planes and performing manual regional shimming, BOLD-CMR was performed in a single mid-ventricular short-axis view at baseline (BL 1-3) and during intracoronary vasodilator infusion into the LCX. Acetylcholine (ACh) was infused in three increasing doses as previously described [16]: 0.1 μg/min (ACh 1), 1 μg/min (ACh 2) and 10 μg/min (ACh 3). Adenosine (Ade) was infused at the following rates: 30 μg/min (Ade 1), 150 μg/min (Ade 2) and 300 μg/min (Ade 3). Measurements were performed in the following sequence: BL 1, ACh 1-3, BL 2, Ade 1-3, and BL 3. At the end of the protocol, we acquired a series of images during first-pass perfusion using a single-shot GRE-EPI sequence after intracoronary injection of 0.05 mmol/kg gadopentetate dimeglumine (Magnevist^®^, Bayer, Germany) for accurately identifying the LCX perfusion territory and confirming the correct position of the intracoronary catheter.

SSFP BOLD-CMR was performed with a T_2_*-sensitive cine SSFP sequence as previously described [15]. Scan parameters were: FOV = 228 × 280 mm; matrix size = 125 × 192; in-plane resolution = 1.8 × 1.6 mm; slice thickness = 5 mm; TR/TE = 5.8 ms/2.9 ms; flip angle = 90°; readout bandwidth = 275 Hz/Px; signal averages = 1; the duration of the typical breath-hold was 15 s. In addition, a segmented multi-echo gradient echo (GRE) sequence was used (echo train length: 8; T_E _= 2.6, 4.8, 7.0, 9.3, 11.5, 13.7, 16.0, and 18.2 ms) using a mono-polar readout. K-space lines from the different echoes were assigned to different images, and the resulting 8 images were used to generate a T_2_* map. Other typical parameters for the multi-echo GRE sequence were: FOV = 225 × 400 mm; matrix size = 109 × 256; in-plane resolution = 2.1 × 1.6 mm; slice thickness = 10 mm; flip angle = 20°; TR = 120 ms; and typical breath-hold duration, 16 s. Reproducibility was assessed by 20 repeated baseline SSFP BOLD-CMR acquisitions in each of three subjects.

### Data Analysis

**Figure 1:**
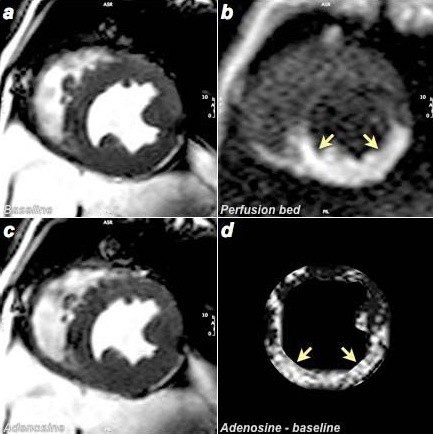
**Examples of BOLD-CMR images a) at baseline; c) during infusion of 0.3 mg/min adenosine (Ade 3)**. The perfusion territory of the LCX after intracoronary injection of gadopentetate dimeglumine is shown in b) (arrows). Panel d) shows the myocardial signal intensity of baseline subtracted from that during adenosine. The higher values in the LCX territory reflect the signal intensity increase during in the LCX perfusion territory as induced by adenosine injection.

CMR images were analyzed using certified software (cmr^42^, CIRCLE Cardiovascular Imaging Inc., Calgary, Canada). A region of interest (ROI) was drawn around the LCX perfusion territory, which was clearly identified on the perfusion images as contrast was injected from the infusion catheter placed in the LCX (see Figure 1b). This ROI was then copied to all other images at the same slice location with careful adjustments to account for any mismatches. Myocardial signal intensity in SSFP BOLD-CMR images and T_2_* values were assessed for each protocol stage. The SSFP BOLD-CMR signal intensity was calculated for each of the 20 images obtained throughout the cardiac cycle and then averaged. T_2_* maps were generated by fitting a mono-exponential function of TE through the T_2_*-weighted signal intensities.

Coronary blood flow was recorded from the Doppler flow probe (ml/min). Intra-arterial blood pressure was monitored continuously and the rate-pressure product (RPP) was calculated from heart rate and systolic pressure.

### Statistical Analysis

Continuous variables with normal distribution were summarized as mean ± standard error of the mean. Means at each level of drug infusion were analyzed with repeated measures ANOVA and post-hoc comparisons, using paired *t*-tests with Bonferroni adjustments (SPSS 16 for Macintosh, SPSS Inc, Chicago, USA). Correlation analysis was done (SIGMAPLOT, Windows version 10, Systat Software, Inc. San Jose, CA, USA) by linear regression analysis or by regression analysis using a 3-parameter, exponential-rise equation that asymptotically approached a maximum.

Sample-size calculation was performed aiming for the detection of a 200% increase in flow, which was considered to be relevant in assessment of myocardial perfusion reserve, with 80% power and 95% significance and based on results of previous studies with a similar experimental setting[11,17].

## Results

### Procedure

**Figure 2:**
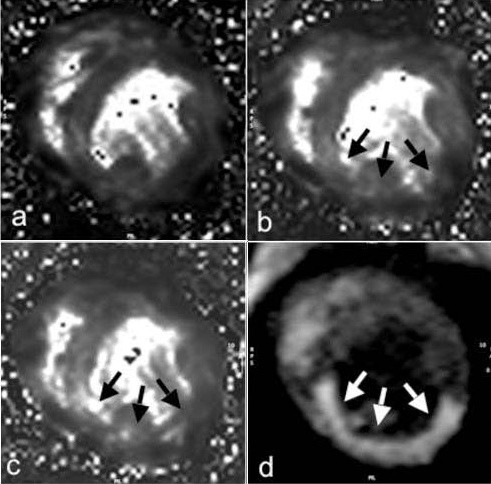
Examples of T_2_* maps at a) baseline, b) during LCX infusion of 10 μg/min acetylcholine (ACh 3) and c) during infusion of 0.3 mg/min adenosine (Ade 3). The perfusion territory of the LCX after intracoronary injection of gadolinium is shown in d) and indicated with white arrows.

The drug-infusion protocol was completed and the perfusion territory of the LCX was identified in all 7 animals (examples see Figure 1b and 2d). In three animals, the adenosine perfusion protocol was shortened because of difficulties during the surgical or interventional procedure. One of the protocol stages in two of these three animals and two protocol stages in the third could not be performed. In one dog, the coronary sinus catheter dislocated after the baseline study and could not be relocated, so blood gas analysis from the coronary sinus was not possible. In another dog, a different SSFP BOLD-CMR sequence was used and only T_2_* data was included in the analysis.

### Coronary Vasodilation Experiments

**Figure 3:**
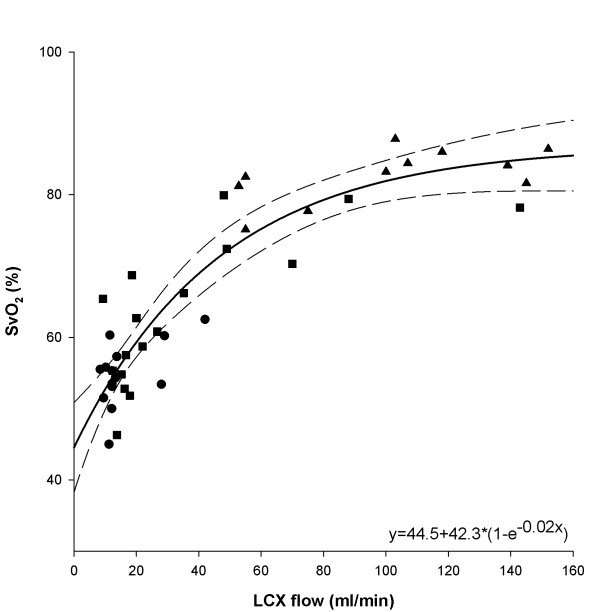
**Correlation of absolute oxygen saturation (SvO_2_) in the coronary sinus (CS) and blood flow in the LCX (n = 7)**. Regression lines (dashed lines) are shown. Baseline, Adenosine and acetylcholine induced changes in flow represented as circles, squares and triangles, respectively. LCX: Left circumflex coronary artery.

Throughout the experiments, there were no significant changes in heart rate, blood pressure and RPP when compared to baseline (Table 1). Drug-induced vasodilation significantly increased LCX blood flow, whereas there was no significant difference between the two repeated baseline measurements (Table 1). There was a dose-dependent increase in blood flow, which was accompanied by progressively smaller increases in coronary sinus oxygen saturation (SvO_2_). Using the 3-parameter, exponential-rise equation, SvO_2 _correlated with LCX blood flow (r^2 ^= 0.84, *P *< 0.001) with SvO_2_approaching 87% (Figure 3). Mean arterial oxygen saturation was 96.7 ± 1.2%.

**Table 1:** Physiological parameters at each experimental level

	BPsys (mmHg)	HR (min-1)	RPP (mmHg/min)	Flow (%)	SvO2 (%)
**BL1**	95.9 ± 2.2	72.0 ± 3.9	6865 ± 276	100.0 ± 0	0 ± 0
**Ach1**	95.6 ± 2.5	69.9 ± 3.2	6650 ± 258	127.7* ± 4.6	3.6 ± 2.3
**Ach2**	98.3 ± 1.7	67.3 ± 3.9	6598 ± 263	186.2* ± 13.3	5.3 ± 2.1
**Ach3**	99.1 ± 2.8	65.6 ± 3.5	6466 ± 282	309.7* ± 29.8	15. 7* ± 0.9
**BL2**	98.5 ± 3.0	67.2 ± 3.5	6577 ± 232	107.5 ± 5.2	5.2 ± 2.0
**Ade1**	97.7 ± 2.3	73.0 ± 3.2	7123 ± 304	817.9* ± 122.1	31.3* ± 2.6
**Ade2**	95.6 ± 4.1	73.4 ± 2.7	7018 ± 410	410.4* ± 64.7	28.9* ± 1.2
**Ade3**	99.0 ± 1.5	74.0 ± 8.7	7352 ± 981	532.2* ± 56.7	31.5* ± 0.6
**BL3**	94.6 ± 2.2	71.6 ± 2.3	6769 ± 259	117.2 ± 6.4	4.4 ± 1.4

p < 0.05 compared to baseline

Mean values and SEM for systolic blood pressure (sysBP), heart rate (HR), rate-pressure product (RPP), flow in the left circumflex coronary artery (flow) relative to baseline 1 and change in coronary sinus oxygen saturation (SvO_2_) from baseline 1 at the different experimental levels (BL 1-3: baseline, ACh 1-3: intracoronary acetylcholine infusion/increasing doses, Ade 1-3: intracoronary adenosine infusion/increasing doses; detailed drug doses in the text). *: p < 0.05 vs. baseline (BL1).

### Oxygenation-sensitive CMR

SSFP BOLD-CMR consistently provided very good image quality (Figure 1). Although image quality of T_2_* maps was consistent, the images showed distortion and susceptibility artifacts, especially with respect to the infero-lateral wall (Figure 2). Representative images for baseline, high-dose ACh and/or high-dose Ade are shown in Figures 1a, c and 2a, b and 2c. After subtracting baseline image signal intensity, changes of signal intensity were visually apparent (Shown for cine SSFP in Figure 1d). The baseline reproducibility of myocardial BOLD-CMR signal intensity in the LCX perfusion territory was excellent (SEM 0.3%). T_2_* measurements showed an SEM of 6.8% at baseline and 1.1% during the protocol.

**Figure 4:**
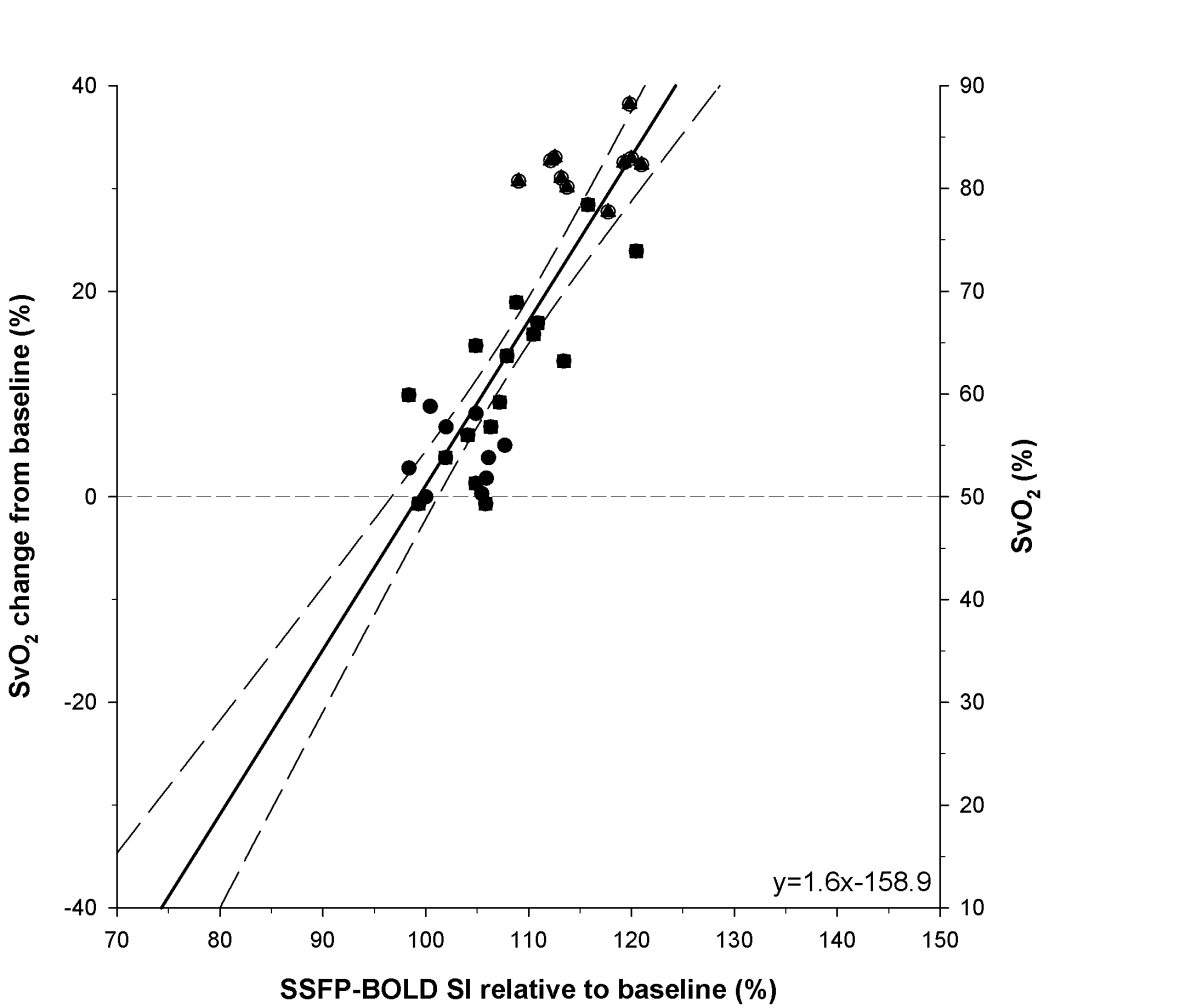
**Linear correlation of SvO_2 _changes from baseline values versus myocardial BOLD-CMR signal intensity relative to baseline (n = 6)**. Regression lines (dashed lines) are shown. Baseline, adenosine and acetylcholine induced changes in flow represented as circles, squares and triangles, respectively. Figure 4 includes the results of the analyses of extrapolated lines. See text. LCX = left circumflex coronary artery.

To see how well CMR tissue measures of oxygenation might predict coronary sinus SvO_2_, we plotted the change in SvO_2 _versus myocardial signal intensity (Figure 4) both expressed as percentages, relative to their respective baseline values. Coronary sinus SvO2 was linearly and strongly correlated with myocardial signal intensity in the SSFP BOLD-CMR images (r2 = 0.74, P < 0.001). T2* changes were relatively larger but showed increased variability (r2 = 0.34, p < 0.001).

## Discussion

Our *in vivo *study indicates that changes in BOLD CMR signal intensity can be used to predict changes in myocardial oxygenation (as indicated by changes in coronary sinus oxygen saturation) accurately, and with a precision that promises to make the technique clinically useful.

To our knowledge, this is the first *in vivo *study to compare oxygenation-sensitive CMR using a state-of-the-art SSFP sequence against simultaneous coronary venous blood sampling. Similar studies have been done using T_2_* mapping for assessing blood oxygenation[18]. Our results showed an excellent linear correlation of coronary sinus oxygen saturation with CMR signal intensity. T_2_* mapping was more sensitive to oxygenation changes but showed more variability. The fact that BOLD CMR shows a linear correlation to coronary sinus oxygen saturation is in agreement with the expected proportionality between the BOLD effect and the absolute tissue content of deoxygenated hemoglobin[13,19,20]. Importantly, a previous theoretical study by Dharmakumar et al. demonstrated that the BOLD signal is blood volume-independent[21].

As shown in Figure 3, the relationship between coronary sinus SvO_2 _and LCX flow can be described using a 3-parameter, exponential-rise equation. With constant myocardial oxygen consumption (i.e., constant RPP), coronary sinus SvO_2 _approached an asymptotic value (87%) as the LCX was over-perfused with near-saturated (97%) arterial blood under the influence of coronary vasodilators (ACh and Ade).

We chose to use both acetylcholine and adenosine to demonstrate that this technique was sensitive to both endothelium-dependent and -independent vasodilation. The same relationship between myocardial BOLD-CMR signal intensity, coronary blood flow, and SvO_2 _was observed for ACh- and Ade-induced flow changes (see Figures 3 and 4). This also excludes a significant impact of nitric oxide on myocardial oxygen consumption in acetylcholine-induced vasodilation, as previously speculated[22]. Therefore, SSFP BOLD-CMR appears suitable for studies with both, endothelium-dependent as well as endothelium-independent vasodilation.

In our study, we uncoupled myocardial oxygen consumption from myocardial blood flow by infusing an intracoronary vasodilator that increased blood flow but not heart rate, blood pressure, or RPP (Table 1), which is a surrogate marker for myocardial oxygen consumption[23]. Thus, our model allowed for a selective assessment of perfusion-induced changes while keeping myocardial oxygen consumption stable.

### Potential for detection of myocardial ischemia

Obviously, it would have been of great interest to be able to follow this relationship into the ischemic range but, unfortunately, our attempts to limit LCX flow were not successful in this experimental preparation. However, the linearity and highly correlated nature of the relation between SvO_2 _and CMR signal intensity suggest that some extrapolation into the ischemic range would be justified. Based on the control values of LCX flow and the correlations we obtained (see Figure 3), we estimated that the normal average value of coronary sinus SvO_2 _at baseline was 50% and that the maximum value was ~90%. Accordingly, we used these values (see right-hand ordinate in Figure 4) to express SvO_2 _relatively, as the change from this control value in % SvO_2_. Thus, we were able to plot changes in coronary sinus SvO_2 _versus relative value of BOLD CMR signal intensity (Figure 4) throughout the range of LCX flows that we observed and extrapolate into the ischemic ranges (note the extrapolation of the linear regression line). Further experimental and clinical studies should be undertaken to test the accuracy and utility of BOLD-CMR in the presence of myocardial ischemia.

### Comparison to Previous Studies

The effects of ACh and Ade on coronary flow shown in this study are within the range of previously reported studies[16,17,24]. Foltz et al. found similar changes in coronary venous oxygen saturation by intracoronary adenosine infusion in the porcine LAD[17]. The corresponding changes in T_2 _they found during maximal vasodilation were in the range of the T_2_* changes we observed, although our T_2_* mapping technique did not provide the same consistency. This may be due to the differences in MR techniques and/or differences in coronary territories assessed (LAD vs. LCX). In our protocol, we included the inferolateral wall, which is particularly susceptible for off-resonance artifacts in BOLD-sensitive CMR[4]. In a similar experimental approach, Fieno et al. could also demonstrate a close correlation of myocardial perfusion and BOLD-sensitive CMR[11]. They used a different BOLD-sensitive CMR technique, which resulted in relatively higher SI changes (up to 30%), which, however, were linearly correlated to myocardial flow, whereas we found an exponential correlation that matches the T_2_* and SvO_2 _changes. We believe that this difference might be explained by T_2 _and T_1 _interference in T_2_-prepared SSFP imaging since the acquisition follows a T_2_-preparared SSFP imaging since the acquisition follows a T_2_-preparation period followed by signal readout during which T1 recovery is also expected to occur. Finally, in our method, the relatively short breath hold of less than 15 seconds is an advantage.

### Clinical Implications

Besides its apparent utility for clinical research, BOLD-CMR appears particularly useful for the clinical assessment of coronary perfusion reserve as used for the detection of relevant coronary artery stenoses. Since BOLD-CMR provides a biomarker (Hb oxygenation) instead of surrogate markers, it may overcome many limitations of currently used diagnostic tools. Furthermore, it can be applied as part of a comprehensive CMR study including cardiac morphology, function, coronary anatomy, ischemia, and tissue pathology.

### Limitations

The critical element in our experimental design is that SvO_2 _should reflect the degree of oxygenation of the LCX myocardium that we evaluated by BOLD SI. We cannot rule out the possibility that there was some admixture by blood draining from areas other than the LCX-perfused myocardium. However, the LCX is the dominant coronary artery in the dog and its course parallels that of the coronary sinus. Furthermore, as pharmacological vasodilatation increased LCX flow, SvO_2 _approached 87% -- only 10% below arterial saturation -- suggesting that LCX drainage was overwhelmingly dominant.

## Conclusions

Oxygenation-sensitive CMR using a T_2_*-sensitive SSFP BOLD sequence allows for non-invasive assessment of changes of myocardial oxygenation *in vivo*; specifically changes in BOLD signal are impressively proportional to changes in coronary sinus oxygen saturation. Further studies to assess the feasibility and accuracy of this technique in clinical settings are warranted.

## Abbreviations

BOLD: Blood-Oxygen-Level-Dependent; CMR: Cardiovascular Magnetic Resonance; FOV: Field of view; Fr or F: French; GRE-EPI: GRadient Echo-Echo Planar Imaging; LCX: Left circumflex coronary artery; MRI: Magnetic resonance imaging; Px: Pixel; SSFP: Steady-state-free-precession; SvO_2_: Venous oxygen saturation

## Competing interests

The research was supported by the *Husky Energy Research Program for the Early Detection of Cardiovascular Risk*. During the experiments, Matthias Vöhringer was supported by a research scholarship from Robert-Bosch-Foundation, Germany and was a Canadian Institute of Health Research (CIHR) strategic training fellow in TORCH (Tomorrows Research in Cardiovascular Health Professionals), Rohan Dharmakumar was supported by American Heart Association (SDG 0735099N) and National Institutes of Health (HL 091989), and Jordin Green was a full-time employee with Siemens Canada Limited; Matthias Friedrich is a scientific advisor and stockholder of Circle Cardiovascular Imaging Inc.

## Authors' contributions

MV has performed the invasive experiments, collected the data, performed the evaluation and was significantly involved in writing the manuscript. JAF was responsible for the experimental setup of the experiments and in gathering and documenting the data. She participated in the scanning procedures and was significantly involved in writing the manuscript. JDG has modified the CMR sequences for our study. He was significantly involved in all scanning procedures and in the preparation of the manuscript. RD developed the CMR sequence and was involved in designing the CMR experiments and in writing the manuscript. JJW was involved in evaluation of the data related to analyses of coronary blood flow and significantly involved in preparing the figures. JVT was significantly involved in designing the experiments and the protocol and in writing the manuscript. MGF was responsible for the overall concept and was significantly involved in the study and protocol design. He was also significantly involved in writing the manuscript.
